# Task Sensitivity in L2 English Speakers’ Syntactic Processing: Evidence for Good-Enough Processing in Self-Paced Reading

**DOI:** 10.3389/fpsyg.2020.575847

**Published:** 2020-09-11

**Authors:** Maryann Tan, Anouschka Foltz

**Affiliations:** ^1^Centre for Research on Bilingualism, Faculty of Humanities, Stockholm University, Stockholm, Sweden; ^2^Institute of English Studies, University of Graz, Graz, Austria

**Keywords:** sentence processing, second language (L2), task effects, syntactic ambiguity, good-enough processing

## Abstract

Native (L1) and second-language (L2) sentence processing can sometimes be shallow. A Good-Enough approach suggests that speakers may engage in shallow processing if the task permits. This study tests English native speakers and native Chinese L2 learners of English to explore whether different task demands affect their sentence processing. In a self-paced reading task, participants read globally or temporarily ambiguous sentences with relative clauses preceded by a matrix clause containing two noun phrases (NPs). Comprehension questions modulated task difficulty: Half the participants received comprehension questions that probed their interpretation of the relative clauses whereas the remaining half received superficial questions unrelated to the relative clauses. Task difficulty affected reading times at the point of disambiguation for both L1 and L2 participants. Additionally, participants’ attachment choices for globally ambiguous sentences were consistent with reading times of the disambiguating region in both L1 and L2 readers. The results suggest that both L1 and L2 syntactic processing is modulated by the task at hand. We argue for a similar treatment of shallowness for L1 and L2 speakers in models of sentence processing, along the lines of the Good-Enough approach to language processing.

## Introduction

Cognitive processes are often sensitive to the task at hand. For example, task manipulations have led to significant effects on sentence processing in both native and non-native speakers, sometimes with differing results for native compared to non-native speakers (e.g., Kamide and Mitchell, 1997; Williams, 2006; Swets et al., 2008; Leeser et al., 2011; Prego and Gabriele, 2014; McManus and Marsden, 2017; Shiu et al., 2018). However, it is only relatively recently that task demands have explicitly been incorporated into models of sentence processing (Ferreira et al., 2002; Ferreira and Patson, 2007). In this study, L1 and L2 speakers of English read globally and temporarily ambiguous sentences and answered comprehension questions about the sentences in one of two task conditions: Easy questions only required a superficial reading of the sentence in order to correctly answer the question, while more challenging questions probed participants’ interpretation of the ambiguity. Task difficulty might affect online sentence processing by inducing a deeper structural analysis for the more difficult questions and more shallow processing when answering more superficial questions.

### Syntactic Parsing Principles

Much of the early debate in the field of sentence processing focused on issues of primacy of syntactic parsing (e.g., Ferreira and Clifton, 1986) and which syntactic parsing principles could best explain how complex sentences are read in general. In the case of ambiguous relative clause attachments – the focus of the current paper – several parsing models [e.g., the late closure principle, Frazier (1987); the recently principle, Gibson et al. (1996); and the Unrestricted Race model (Race), van Gompel et al., 2001] were described in light of evidence from experimental work on native speaker reading behavior. When L2 sentence processing research applying online methods grew in number, such structural parsing principles became the benchmark from which to measure whether L2 speakers could acquire the purported parsing intuitions that native speakers possess (cf. Clahsen and Felser, 2006b).

One aspect that established parsing models tend to ignore is the outcome of comprehension when reading syntactically challenging sentences. This fact was emphasized by Ferreira (2003) in reaction to the narrowing focus of the field on the question of whether or not sentence parsing operates independently of other non-syntactic information that could influence sentence processing. Discussed in the context of L2 research, this primacy of structural parsing has become the deciding factor for native-like attainment by L2 speakers (see Clahsen and Felser, 2006b, and the studies discussed within) where evidence for native-like structure-based parsing is interpreted as a deep parse and a lexical-semantic-driven analysis, a shallow parse.

In classic models of sentence processing it is implicitly assumed that representations of meaning built on syntactic analysis are accurate and complete. But even native readers may fail to interpret certain sentence structures accurately, as demonstrated with garden-path sentences like *While Anna bathed the baby played in the crib* (Christianson et al., 2001) or non-canonical passive constructions (Ferreira, 2003) with atypical agent-patient relationships like *The dog was bitten by the man*. In the first example, follow-up comprehension questions revealed that native speakers interpreted *the baby* as both the patient of the subordinate clause and the subject of the main clause. In the second, native speakers wrongly interpreted *the dog* as the agent and *the man* as the patient in response to comprehension questions probing their thematic role assignment, presumably because common knowledge informs us that canines are more likely to bite humans than the other way around. These results suggest that even when a correct syntactic parse was performed, readers’ world knowledge may impinge upon interpretations, resulting in what is referred to as a shallow or “good-enough” interpretations (Ferreira et al., 2002; Ferreira and Patson, 2007).

### The Good-Enough Approach in L2 Sentence Processing

In its original conception, the Good-Enough approach was described in light of findings from native speaker interpretations, but given the increasing evidence that L2 speakers largely show similar reading patterns to native speakers as long as they are sufficiently proficient (e.g., Hopp, 2006; Williams, 2006), the Good-Enough model might be well-suited to explain processing strategies in L2 speakers as well.

Recently, researchers have therefore begun to consider the Good-Enough approach to explain L2 comprehension. Lim and Christianson (2013a) hypothesized that L2 learners process language with a combination of semantic-based heuristics and their L2 grammar, i.e., algorithmic processing. Instead of the L2 grammar being qualitatively different from the L1 grammar, the syntactic output in an L2 context may be relatively more fragile and thus more susceptible to the influence of world knowledge (heuristics).

The Korean-speaking learners of English who participated in Lim and Christianson’s (2013a) study verbally translated plausible and implausible auditory sentences like those in (1) from English into Korean in one experiment and from Korean into English in a second experiment.

(1a) *The dog bit the man.* (plausible; active),
(1b) *The man was bitten by the dog.* (plausible; passive),
(1c) *The man bit the dog.* (implausible; active),
(1d) *The dog was bitten by the man.* (implausible; passive).

The study showed that L2 speakers, similar to native speakers in Ferreira (2003), produced a substantial number of “good-enough” translations, where they correctly translated the syntactic structure of the sentence, but reversed thematic roles. For example, *The dog was bitten by the man* was translated as *The man was bitten by the dog*. This suggests that participants achieved a proper parse of the original sentences, but that their translation was influenced by world knowledge (heuristics) which dominated the ultimate interpretation. Lim and Christianson (2013a) argued that both routes of syntactic and semantic analysis are active in L2 processing, but a failure to integrate them results in a translation that was “good-enough” (cf. also Lim and Christianson, 2013b, for similar results). In the reverse condition, where L2 speakers translated analogous sentences from their L1 Korean into L2 English, they made relatively fewer thematic assignment errors in the passive implausible condition (1d) compared to native speakers in the Ferreira (2003) study. The difference between the two studies was that the L2 learners were required to listen and translate the sentences into English, while the native speakers were only required to respond to questions on thematic role assignment. This points to the possibility that the translation task had the effect of increasing speaker effort to build more detailed representations during comprehension, resulting in an interpretation that was more resistant to effects of world-knowledge heuristics.

### Task Difficulty and the Good-Enough Approach

One factor that can force a resolution on conflicting syntactic and semantic interpretations is task difficulty. In a task-driven or goal-dependent framework such as the Good-Enough model (Ferreira et al., 2002; Ferreira and Patson, 2007), readers are assumed to actively use both deep syntactic algorithmic processing and shallow processing (Karimi and Ferreira, 2016; see also Clahsen and Felser, 2017). Because shallow semantic processing is built upon general beliefs about the world, this processing route is not fail-safe in terms of accuracy of interpretation, but tends to operate more quickly than deliberate algorithmic processing. However, the goal of comprehension, i.e., the task that follows the reading exercise, can influence to what extent either parsing route is recruited. If, for example, readers are aware that they require only a superficial understanding of a sentence to answer a comprehension question, they are more likely to make use of shallow parsing heuristics than engage in effortful syntactic analysis.

Few L2 studies have directly manipulated the difficulty of a certain task as a factor, and few have actually analyzed the concomitant offline interpretation results of reading. The current study attempts to find further evidence for Good-Enough processing mechanisms in L2 processing by expanding the design and methods of a study (Swets et al., 2008) on native speaker resolution of ambiguous relative clauses to L2 speakers.

Swets et al. showed that the depth of syntactic processing by native speakers is indeed sensitive to the difficulty of the task at hand. Participants in their study read globally ambiguous and disambiguated sentences like in (2) in a self-paced reading task. One group of participants received questions that probed their interpretation of the relative clause (e.g., *Did the princess scratch in public?*), whereas two other groups answered superficial questions that could be answered without making an attachment decision, i.e., without deciding who scratched in public (e.g., *Was anyone humiliated?*).

(2a) Globally ambiguous:
  The maid of the princess who scratched herself in public was terribly humiliated.
(2b) Disambiguated; high, or N1, attachment:
  The son of the princess who scratched himself in public was terribly humiliated.
(2c) Disambiguated; low, or N2, attachment:
  The son of the princess who scratched herself in public was terribly humiliated.

Replicating previous results by proponents of the Race model (Traxler et al., 1998; van Gompel et al., 2001, 2005), they found that globally ambiguous sentences were read as fast as or faster than temporarily ambiguous sentences. The Good-Enough approach explains these results by suggesting that participants did not make an attachment decision for the globally ambiguous sentences during reading. Rather, they left the relative clause unattached and the parse underspecified, resulting in faster reading times when the comprehension questions were superficial. However, this globally ambiguous sentence advantage was attenuated when readers were asked questions about the relative clause. The globally ambiguous condition was read at similar speeds to the low attachment sentences, while the high attachment sentences were read more slowly. Thus, the Good-Enough approach places importance on reader goals in its account of sentence processing behavior.

In a later specification of the mechanisms underlying Good-Enough processing, Karimi and Ferreira (2016) note the priority of processors to maintain a state of equilibrium. Algorithmic (syntactic) processing is distinguished from heuristic (world knowledge) processing. Comprehenders prioritize a state of equilibrium and will stay in such a state unless they receive signals that mandate deeper processing by algorithmic means. Thus, although both kinds of processing occur during a parse, time, resources, the goals of the comprehender, etc., influence the relative application of these two processing routes. In particular, the relative activation of syntactic and semantic processing can be influenced by factors such as the goals of comprehension, where speakers may engage in shallow parsing if the task at hand allows them to do so. In the ambiguous sentence examples in (2), the complex noun phrase with the two potential antecedents acts as a single entity as comprehenders encounter the reflexive pronoun. A globally ambiguous sentence maintains this equilibrium, but a reflexive pronoun that can only refer to one of the antecedents in the complex noun phrase may force the processor to break up this merged representation, causing disequilibrium and triggering longer algorithmic processing. In this model, attachment decisions are unnecessary until the processor encounters a signal that forces it to process the relative clause in greater depth. Thus, under conditions that do not require or force a thorough reading, Good-Enough parsing can resemble Race parsing in the sentences of the kind employed in this study.

The role of task difficulty was clearly revealed in Swets et al.’s (2008) study: Participants in the superficial-question group read the sentences faster than participants in the relative-clause question group, especially upon encountering the reflexive pronoun *herself* or *himself* (which disambiguates the sentence in the two disambiguated conditions). This suggests that the type of question (i.e., the difficulty of the task at hand) modulates the depth of processing, with shallower processing for superficial questions compared to relative-clause questions.

Swets et al.’s (2008) question-response data support this idea. Participants in the relative-clause question group took longer to respond than participants in the superficial question group. In addition, participants in the relative-clause question group, but not participants in the superficial question group, took longer to respond to questions following globally ambiguous sentences compared to disambiguated sentences. This suggests that participants only attached the relative clause of the ambiguous sentences when asked to do so by the comprehension question. Altogether, the results showed that task difficulty influenced the reading strategies of L1 readers and that processing can be shallow if insufficient information is available for the processor to make a definitive interpretation or if the task at hand does not require a definitive interpretation.

### Relative Clause Attachment

The Good-Enough account assumes that attachment decisions will only be made when necessary, i.e., when the processor is forced to process a relative clause in greater depth. However, the Good-Enough account does not make any predictions about which particular attachment choice readers will make.

Of note for the sentence structures we examine here is the Race model (van Gompel et al., 2001, 2005). Race is non-deterministic, which means parsers can either take an N1 or N2 attachment route. In globally ambiguous sentences, both N1 and N2 interpretations are compatible with the final interpretation as neither conflicts with the reflexive pronoun. In contrast, only one option is compatible in temporarily ambiguous sentences; if the wrong noun phrase was adopted early in the parse, a reanalysis will have to be made, leading to longer reading times. This implies that globally ambiguous sentences should have a reading time advantage over at least one of the disambiguated sentences. That is, if a parser tends to have an N2 bias, it would have to reanalyze disambiguated sentences that conflict with this initial interpretation. But in sentences that do not conflict, reading will be as quick as globally ambiguous sentences. Given that Race allows for variability in attachment choices, it could reasonably explain any attachment preference results to be found in the current study.

Under conditions that do not require or force a thorough reading, Good-Enough parsing can resemble Race in the sentences of the kind employed in this study. However, in questions that probe relative clause interpretation one should expect to find evidence for slower processing overall and slower processing of globally ambiguous sentences, which can manifest in slower reading times or question response times.

Prior studies have focussed intensely on cross-linguistic differences in attachment preferences (see Papadopoulou, 2006, for a review). While low attachment preferences have been found in English, these biases are not necessarily strong (e.g., Carreiras and Clifton, 1993, 1999). The findings on native Chinese speakers have been mixed, reporting either a low attachment preference (Shen, 2006; Kwon et al., 2019) or a high attachment preference (Cai, 2009; Cai, n.d. for Chinese relative clause sentences.

Studies in L2 processing of ambiguous relative clauses typically compare L2 attachment preferences with the preferences of native speakers. A reliable preference for either high or low attachment would suggest that at least one phrase-structure principle (cf. Frazier, 1987; Gibson et al., 1996) is in operation during L2 processing. In contrast, a lack of an attachment preference has been taken as evidence for shallow parsing (Felser et al., 2003; Papadopoulou and Clahsen, 2003). While studies have found clear attachment preferences for native Chinese speakers processing ambiguous relative clause sentences in English, they have also been mixed; both low (Dai, 2015) and high attachment (Witzel et al., 2012) preferences have been reported.

### The Current Study

The current study extends the native-speaker results from Swets et al. (2008) by testing both native and non-native English speakers. In particular, it investigates whether task demands influence the processing of globally ambiguous and disambiguated relative-clause sentences in L1 Chinese learners of English. If the learners change their processing strategies according to their goals, this would support a Good-Enough approach to sentence processing. To investigate task effects, we measured both reading times in a self-paced reading task as well as response accuracy and response times for comprehension questions.

Participants in the current study read both globally ambiguous and disambiguated English relative-clause sentence as in (2) above. Prior studies investigating relative-clause processing by L2 learners have often only compared online reading of two attachment conditions (high and low; e.g., Dussias, 2003; Felser et al., 2003; Papadopoulou and Clahsen, 2003; Witzel et al., 2012). By introducing a third, globally ambiguous condition, this study tests whether L2 learners’ online reading strategies change according to the type of ambiguity.

Task demands were manipulated in the current study as in Swets et al. (2008), i.e., by means of the comprehension questions. One group of participants received comprehension questions probing their relative-clause attachment. The other group received superficial questions that could be answered without attaching the relative clause. The current study differs from Swets et al. (2008) in that our task manipulation is more subtle. Rather than varying the difficulty of comprehension questions for all sentences, we only varied the difficulty of comprehension questions following target sentences, but not following filler sentences. Both L1 and L2 participants may process globally ambiguous sentences and temporarily ambiguous sentences differently under these different task conditions. If their reading strategies change according to task difficulty, even if the difference in task difficulty is subtle, then this would demonstrate the goal-dependent nature of both L1 and L2 processing. In particular, a Good-enough approach would predict shorter reading times for the superficial-question group compared to the relative-clause question group, especially in the disambiguating region of the sentences.

A combination of the reading time data and the question-response time data can also shed light on when the L1 and L2 participants receiving relative-clause questions make attachment decisions. In the case of disambiguated sentences, readers are expected to attach the relative clause when reading the disambiguating reflexive pronoun. In the case of globally ambiguous sentences, a Good-enough approach suggests that readers could delay attachment of the relative clause until the question that probes their attachment. If participants attach the relative clause during online processing of the globally ambiguous relative clause, reading times should be similar for globally ambiguous and disambiguated sentences for those receiving relative-clause questions. But if participants attach the relative clause only when probed through a comprehension question, then question-response times to relative-clause questions should be slower for globally ambiguous sentences than disambiguated sentences, since readers will need to make an attachment decision while answering the question.

A Good-enough approach would further predict speed-accuracy trade-offs within the group of participants receiving relative clause questions. Specifically, participants who engage in deeper processing of the reflexive pronoun should show both longer reading times, i.e., less speed, for the reflexive pronoun and higher question-response accuracy compared to those engaging in more shallow processing.

Finally, this study contributes additional data to the sparse existing literature on L2 attachment preferences by native Chinese speakers. Based on previous studies, we would expect a low attachment preference for English speakers. However, previous attachment preference results for native Chinese speakers reading both English and Chinese sentences are mixed, so that our attachment preference results will contribute to the currently heterogeneous picture of attachment preferences by native Chinese speakers.

The combination of measuring attachment choices, reading times, and question-response times will provide insight into the processes involved in how L2 learners of English derive a final interpretation for globally ambiguous relative-clause sentences.

## Materials and Methods

### Participants

A total of 55 participants took part in the study. The L1 group comprised 27 native British English-speaking participants (21 female, 4 male, 1 other, 1 non-response, mean age = 21.9; *SD* = 4.1) who were undergraduates at Bangor University. Four of them reported knowing no other language, and 23 reported knowledge of another language, but considered their language skills to be poor or good for simple conversation. Three further participants, two with dyslexia and dyspraxia and one fluent bilingual, were excluded from the study. All participants in the L1 group had normal or corrected to normal vision.

The L2 group included 28 native Chinese speakers from China, Taiwan, and Malaysia (17 female and 11 male, mean age = 27, *SD* = 4.85), who lived in different parts of the UK at the time of testing. They all acquired the Beijing dialect of Mandarin either as a mother tongue or through the national schooling system, which had Mandarin as the main medium of instruction. Where their home language was not Mandarin, they either spoke Cantonese (7 participants) or another regional dialect (e.g., Henan dialect, Sichuan dialect). While the Chinese dialects (including Cantonese) vary in tones, vocabulary and syntax, they are believed to have evolved from the same language (Lee and Li, 2013).

All participants in the L2 group began learning English in primary school and did not spend their formative years in an English-speaking community. The mean age of first exposure to English was at 10.33 years (*SD* = 3.08). All L2 participants lived in the United Kingdom at the time of the study. Their average time spent in an immersive environment was 3.69 years (*SD* = 4.50). The L2 participants self-reported their English proficiency in four skill areas (reading, writing, speaking, and understanding) using a 4-point scale with 1 being “barely/not at all” and 4 being “fluent.” The mean self-rating scores are 3.37 (*SD* = 0.48) for reading, 3.22 (*SD* = 0.57) for writing, 3.33 (*SD* = 0.61) for speaking, and 3.48 (*SD* = 0.57) for listening comprehension.

All except one L2 participant had normal or corrected to normal vision. The participant with abnormal vision reported a congenital defect in his right eye, which has rendered it blind since childhood. The participant reported that he had adapted to his monocular vision and experiences no difficulties with reading on-screen.

### Materials

The 36 experimental sentence items and comprehension questions were taken from Swets et al.(2008; see (2) above). Each sentence item, such as *The maid/son of the princess who scratched herself/himself in public was terribly humiliated* [see (2) above], consisted of three relative-clause sentences, a globally ambiguous one [with *maid* and *herself*; see (2a)], one disambiguated to N1 [high attachment, with *son* and *himself*; see (2b)], and one disambiguated to N2 [low attachment, with *son* and *herself*; see (2c)]. In addition, there were two questions for each experimental sentence item, one probing the attachment of the relative clause (e.g., *Did the princess scratch in public?*), and one superficial question that did not require making an attachment choice (e.g., *Was anyone humiliated?*). Half of the questions required a *yes* response and the remaining half required a *no* response (except for relative-clause questions following a globally ambiguous sentence, which gauged attachment preferences). The 72 filler items consisted of sentences with varying grammatical structures followed by *yes*/*no* comprehension questions that required a considerable understanding of the sentences in order to be answered (e.g., the sentence *The students were bored by the teacher* followed by the question *Did the teacher bore the students?*).

Six experimental lists were created, with sentence type as a within-subjects factor and question type as a between-subjects factor. The sentence items were distributed across three lists in a Latin Square design. The filler sentences appeared in a pseudorandom order and ensured that no target sentences would appear consecutively during the experiment. Two versions of these three lists were then created, which only differed in the comprehension questions for target sentences: Target sentences in one version received relative-clause questions and target sentences in the other version received superficial questions. This resulted in a total of six lists. Questions for filler sentences were the same across all six lists.

### Procedure

The main experiment was conducted with E-prime professional software version 2.0 (Psychology Software Tools, 2012) in a quiet environment. Bilingual participants were tested in various quiet locations across the UK. Monolingual participants came to the Bangor University Linguistics Lab for participation. Bilingual participants read the sentences off a Samsung laptop (Intel Core i3-3110M CPU @2.4 GHz) with a 15.6” screen, and monolingual participants read sentences off a Stone desktop (Intel Core 2 Duo CPU e8400 @3 GHz) with a 24” screen. All participants read the sentences in a self-paced, region-by-region, moving window paradigm.

The sentence regions were presented in a phrase-by-phrase manner rather than word-by-word to facilitate easier reading and comprehension for the L2 English speakers tested in the study. Similar studies conducted on upper intermediate L2 speakers have found that a word-by-word division risked yielding uninterpretable data (Pan and Felser, 2011). To allow a direct comparison of the two groups, the monolingual native English speakers received the same phrase-by-phrase sentence presentation for target sentences. However, due to experimental error, the phrase-by-phrase presentation differed across lists for some of the filler sentences. The target experimental items were divided into regions as shown in (3) (cf. Traxler et al., 1998), where slashes indicate region boundaries, and numbers provide the region number. Participants controlled their reading pace and answered questions by pressing designated buttons.

**Table:** 

(3)	*The maid*	/	*of the princess*	/	*who scratched*	/	*herself*	/
	1		2		3		4	
	*in public*	/	*was terribly*	/	*humiliated*	
	5		6		7	

Each trial started with a message telling participants to push the “continue button” to start the trial. Upon pressing the continue button, a series of dashes would appear, indicating the number of regions. With the first button press, the first dash would be replaced with the words in that region of the sentence. With each subsequent button press, the next dash would be replaced with the words in that region and the words of the preceding region would revert back to a dash. Upon completing the final region, another press of the “continue button” revealed the comprehension question. Participants underwent a practice session with 10 sentences and questions to familiarize themselves with the reading process. The practice sentences and questions were the same for both groups.

Following the main experiment, each participant performed two working memory tests: a digit span backward test and a letter-number sequencing test taken from the Wechsler Adult Intelligence Scale—fourth edition (WAIS-IV; Wechsler, 2008). Results from these tests are beyond the scope of the current study, but we mention the tests here for reasons of transparency. Lastly, participants filled in a language background questionnaire.

### Data Preparation

Twelve trials (0.6% of the data) were missing due to E-Prime crashing unexpectedly near the end of the experiment for four participants. Since only a few trials were affected in each case and in total, and since linear mixed effects models can deal with missing data points, we decided to keep these participants. For the remaining trials, we excluded outliers for reading times using a two-step approach. Both steps were done separately for each region and participant group. First, extreme outliers were identified through a histogram and excluded. We then excluded all reading times that were two standard deviations above or below the mean for a given participant and a given item. Overall, 4.55% of region 4 and 4.09% of region 5 reading times were excluded. Outliers for question-response times were excluded in the same manner: A histogram first identified the extreme outliers to be excluded. Then, all question-response times that were two standard deviations above or below the mean for a given participants and a given item were excluded. Overall, 4.85% of all question-response times were excluded. The data and scripts for all analyses are available on the Open Science Framework at https://osf.io/4szpx/.

## Results

### Reading Times

Our statistical analyses focus on the disambiguating region (region 4) and the possible spill-over region (region 5; Jiang, 2012; cf. also Carreiras and Clifton, 1999; Dussias, 2004; Dussias and Sagarra, 2007; Jegerski, 2018). We analyzed reading times separately for these regions using linear mixed effects modeling and model comparisons (cf. Baayen, 2008). Initial models included sentence type (ambiguous, N1 attached, N2 attached), question type (relative clause, superficial), participant group (L1, L2), and all interactions as fixed effects. We additionally included region length (number of characters) and its interactions with other fixed factors in the initial model for region 5 to account for reading time differences due to region length. This was not necessary for region 4 (*himself/herself*), which had a constant region length of seven characters. Sum-coding was used for ANOVA-style main effects. The random effects structure included random intercepts for participant and item, and by-participant and by-item random slopes for all within-participant factors. Redundant random and fixed effects or interactions (i.e., whose exclusion did not significantly decrease model fit) were removed from the initial model (cf. Baayen, 2008). This model comparison yielded the final models reported here. Furthermore, we report the marginal and conditional R^2^_GLMM_ values for generalized linear mixed effects models to gauge effect sizes throughout. The marginal R^2^_GLMM_ value expresses the variance explained by a model’s fixed factors, and the conditional R^2^_GLMM_ expresses the variance explained by a model’s fixed and random factors (Nakagawa and Schielzeth, 2013; Johnson, 2014; Nakagawa et al., 2017).

#### Disambiguating Region

**FIGURE 1:**
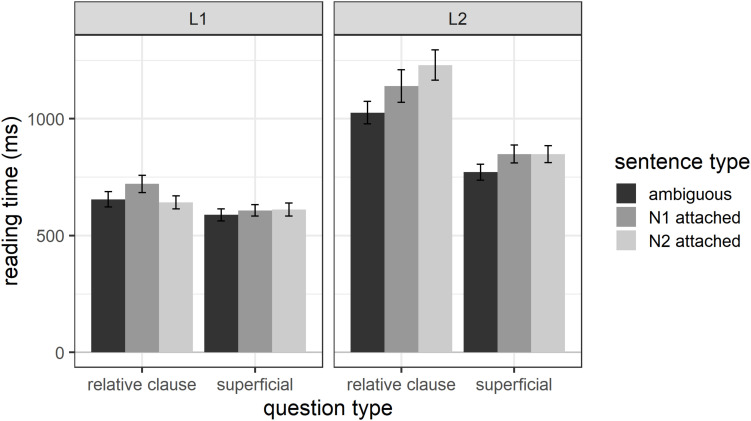
Reading times for region 4 as a function of question type, sentence type, and participant group.

Overall, L1 participants had average reading times of 633 ms (*SD* = 361) and L2 participants of 975 ms (*SD* = 658) for region 4 – the disambiguating region. Figure 1 shows the reading times for each sentence type, question type, and participant group for region 4. The figure shows that, numerically, N1-attached sentences are processed the slowest in the L1 group, whereas N2-attached sentences are processed the slowest in the L2 group. Table 1 shows the results of the final mixed effects model (which included random intercepts for participant and item) for the disambiguating region. The results revealed main effects of question type, sentence type and participant group. In addition, the sentence type by participant group interaction was significant. All other fixed factors were excluded from the model during model comparisons.

**TABLE 1:** Results of the final mixed-effects model for the disambiguating region.

Fixed factor	Estimate	*SE*	*t-*Value	*p-*Value
Question type	–100.47	38.37	–2.62	=0.012
Sentence type	29.88	10.15	2.94	=0.003
Participant group	167.97	38.37	4.38	<0.001
Sentence type × participant Group	28.86	10.18	2.836	=0.005

The main effect of question type shows that participants who received superficial questions read the disambiguating region significantly faster than participants who received relative-clause questions. To further explore the main effect of sentence type, we performed *post hoc* analyses using the emmeans() function (Lenth, 2019) in R. Overall, participants processed the reflexive pronoun significantly faster in ambiguous sentences compared to sentences disambiguated toward N1 (estimate = −79.4, *SE* = 25.1, *t* = −3.16, *p* = 0.005) and toward N2 (estimate = −71.9, *SE* = 24.9, *t* = −2.89, *p* = 0.011). There was no reliable difference in reading times for the disambiguating region between sentences disambiguated toward N1 and N2 (estimate = 7.4, *SE* = 25.1, *t* = 0.295, *p* = 0.953). The main effect of participant group shows that reading times for the L1 participants were, as would be expected, significantly faster than for the L2 participants.

The final model has a marginal R^2^_GLMM_ of 0.13 and a conditional R^2^_GLMM_ of 0.39, suggesting that relatively little, i.e., 13%, of the variance in reading times can be explained through the fixed effects in the model and 39% can be explained through the fixed and random effects, such that the random effects structure contributes slightly more to the variance in reading times than do the fixed effects.

To explore the significant sentence type by participant group interaction, we performed *post hoc* tests for sentence type using the emmeans() function separately for the L1 and L2 groups. The results revealed that L1 participants processed the disambiguating region for sentences disambiguated toward N1 numerically more slowly (mean = 658 ms, *SD* = 373) than sentences disambiguated toward N2 (mean = 625 ms, *SD* = 354) and globally ambiguous sentences (mean = 618 ms, *SD* = 358). However, in both cases this difference did not reach significance (N1- vs. N2-attached: estimate = 47.92, *SE* = 22.3, *t* = 2.15, *p* = 0.081; N1-attached vs. globally ambiguous: estimate = −50.90, *SE* = 22.4, *t* = −2.28, *p* = 0.060). There were also no significant differences in reading times for the globally ambiguous sentences compared to N2-attached sentences (estimate = −2.98, *SE* = 22.1, *t* = −0.14, *p* = 0.990).

In contrast, L2 participants read the disambiguating region significantly faster for ambiguous (mean = 896 ms, *SD* = 549) compared to N1-attached sentences (mean = 994 ms, *SD* = 718; estimate = −107.9, *SE* = 43.7, *t* = −2.47, *p* = 0.037) as well as ambiguous compared to N2-attached sentences (mean = 1036 ms, *SD* = 691; estimate = −143.3, *SE* = 43.4, *t* = −3.30, *p* = 0.003). There was no significant difference in reading times between the N1- and N2-attached sentences (estimate = −35.4, *SE* = 43.7, *t* = −0.811, *p* = 0.697).

#### Spill-Over Region

**FIGURE 2:**
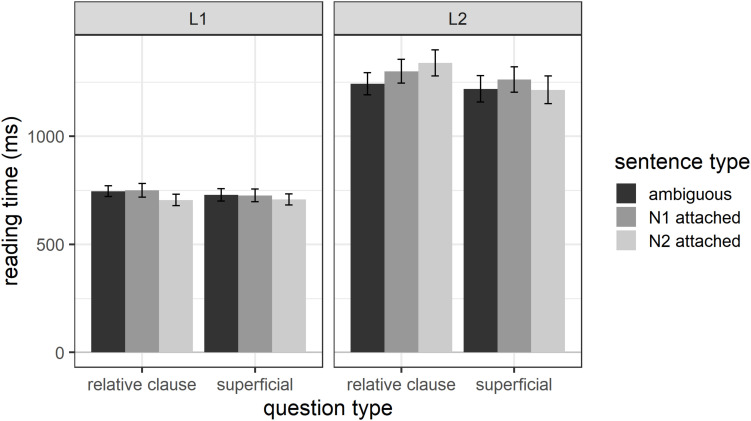
Reading times for region 5 as a function of question type, sentence type, and participant group.

Overall, L1 participants had average reading times of 726 ms (*SD* = 348) and L2 participants of 1263 ms (*SD* = 741) for region 5 – the spill-over region. Figure 2 shows the reading times separately for each sentence type, question type, and participant group for region 5. The figure shows substantially faster reading times for L1 compared to L2 English speakers.

The results for the final mixed effects model (which included random intercepts for participant and item and by-participant random slopes for word length) for the spill-over region reveal three significant effects. There was a significant main effect of participant group (estimate = 270.5, *SE* = 36.4, *t* = 7.432, *p* < 0.001), with L1 participants reading the spill-over region significantly faster than L2 participants, and a significant main effect of region length (estimate = 190.1, *SE* = 30.7, *t* = 6.197, *p* < 0.001), with longer regions taking longer to read. There was also a significant participant group by region length interaction (estimate = 126.3, *SE* = 17.3, *t* = 7.317, *p* < 0.001), due to region length having a larger effect on L2 participants’ reading times than L1 participants’ reading times. In other words, an additional character leads to a larger increase in reading times for the L2 group compared to the L1 group. Importantly, there were no effects involving question type or sentence type, suggesting that any effects of the main experimental manipulations do not carry over into the spill-over region. The final model has a marginal R^2^_GLMM_ of 0.34 and a conditional R^2^_GLMM_ of 0.59, suggesting that 34% of the variance in reading times can be explained through the fixed effects in the model and 59% through the fixed and random effects.

### Responses to Questions

#### Response Accuracy

Table 2 shows the question-response accuracy for all sentence types and question types for the L1 and L2 groups. Since the relative-clause questions probed the attachment of the relative clause (see below for attachment preference results), there are no correct or incorrect responses for globally ambiguous sentences.

**TABLE 2:** Question-response accuracy as percent correct (*SD*) for each sentence and question type for the L1 and L2 groups.

		Relative clause	Superficial
L1	Ambiguous	NA	96.6 (0.18)
	N1 attached	72.0 (0.45)	96.6 (0.18)
	N2 attached	81.8 (0.39)	95.1 (0.22)
L2	Ambiguous	NA	85.0 (0.36)
	N1 attached	70.2 (0.46)	86.3 (0.34)
	N2 attached	62.5 (0.49)	89.9 (0.30)

We used a mixed logit model, which is appropriate for binary responses, to analyze response accuracy (cf. Baayen, 2008). Globally ambiguous sentences were excluded from the analysis. The initial model included sentence type (N1 attached, N2 attached), question type (relative clause, superficial), participant group (L1, L2), and all interactions as fixed effects. The random effects structure included random intercepts for participant and item, and by-participant and by-item random slopes for the within-participant factor sentence type. Redundant random and fixed effects or interactions were removed from the initial model as described above.

The final model included no random factors, and the fixed factors are shown in Table 3. The results revealed a reliable main effect of question type, such that superficial questions had reliably higher accuracy rates than relative-clause questions. In addition, there was a reliable main effect of participant group with significantly higher accuracy rates for L1 compared to L2 speakers. Finally, the sentence type by question type by participant group three-way interaction was significant.

**TABLE 3:** Results of the final logit model for response accuracy.

Fixed factor	Estimate	*SE*	*t-*Value	*p-*Value
Sentence type	0.01	0.09	0.16	=0.874
Question type	0.81	0.09	8.84	<0.001
Participant group	–0.42	0.09	–4.48	<0.001
Sentence type × question type	–0.03	0.09	–0.35	=0.728
Sentence type × participant group	–0.01	0.09	–0.07	=0.942
Question type × participant group	–0.15	0.09	–1.58	=0.113
Sentence type × question Type × participant group	0.21	0.09	2.27	=0.024

To explore the significant three-way interaction, we performed *post hoc* tests separately for participant groups and question type. The results are shown in Table 4 and show that accuracy for N1 and N2 attached sentences did not differ significantly for any of the four *post hoc* comparisons. However, L1 participants receiving relative-clause questions were numerically more accurate in answering questions after their preferred N2-attached sentences compared to the dispreferred N1-attached sentences, and this difference just failed to reach significance.

**TABLE 4:** *Post hoc* comparisons for response accuracy for comprehension questions following N1- and N2-attached sentences.

Subset	Estimate	*SE*	*t-*Value	*p-*Value
L1 relative clause questions	−0.56	0.29	−1.95	=0.051
L1 superficial questions	0.42	0.54	0.78	=0.438
L2 relative clause questions	0.35	0.23	1.50	=0.134
L2 superficial questions	−0.34	0.34	−1.01	=0.314

We performed an additional analysis to test for speed-accuracy trade-off effects in the participants receiving relative clause questions. For this additional analysis, we again used a mixed logit model to analyze response accuracy. Globally ambiguous sentences as well as participants receiving superficial questions were excluded from the analysis. The initial model included sentence type (N1 attached, N2 attached), participant group (L1, L2), reading time for the disambiguating region (numeric), question-response time (numeric), and all interactions as fixed effects. The random effects structure included random intercepts for participant and item, and by-participant and by-item random slopes for the within-participant factors sentence type, reading time, and question-response time. Redundant random and fixed effects or interactions were removed from the initial model as described above. The final model included random slopes for participant and item, all four fixed effects, and the reading time by participant group and sentence type by participant group interactions. In line with our main analysis above, there was no significant effect of sentence type (estimate = 0.04, *SE* = 0.1, *t* = 0.386, *p* = 0.699) on response accuracy. In addition, the analysis confirmed the above main effect of participant group on response accuracy (estimate = −0.40, *SE* = 0.2, *t* = −2.503, *p* = 0.012), and the significant sentence-type by participant group interaction (estimate = −0.3, *SE* = 0.1, *t* = −2.935, *p* = 0.003) is consistent with the three-way interaction found above. Importantly, we found a significant main effect of reading times for the disambiguating region on accuracy (estimate = 0.5, *SE* = 0.2, *t* = 2.355, *p* = 0.019), suggesting that there is indeed a speed-accuracy trade off with less speed, i.e., longer reading times in the disambiguating region, relating to more accuracy for participants receiving relative clause questions. Furthermore, we found a main effect of question response time on accuracy (estimate = −0.2, *SE* = 0.1, *t* = −2.228, *p* = 0.026), suggesting that increased response time relates to less accuracy. It is most likely that participants took longer to answer and were less accurate for questions they thought were more difficult compared to less difficult questions. Finally, we found a significant reading time by participant group interaction (estimate = −0.5, *SE* = 0.2, *t* = −2.362, *p* = 0.018), which we do not explore further here. The final model has a marginal R^2^_GLMM_ of 0.09, suggesting that only 9% of the variance in response accuracy can be explained through the fixed effects in the model.

#### Attachment Preferences

Our analysis of participants’ attachment preferences is restricted to the ambiguous sentences and participants who received relative-clause questions. Overall, L1 speakers chose N2 attachment 60.84% (*SD* = 48.98) of the time, whereas L2 speakers chose N2 attachment 45.24% (*SD* = 49.92) of the time. A Chi-square test showed reliably more N2 attachment compared to N1 attachment decisions for L1 speakers (χ^2^ = 6.72, *df* = 1, *p* = 0.010), but not for L2 speakers (χ^2^ = 1.52, *df* = 1, *p* = 0.217).

We further ran mixed logit models (cf. Baayen, 2008), which are appropriate for binary responses, to analyze attachment preferences. The initial model included participant group (L1, L2) as fixed effect. The random effects structure included random intercepts for participant and item. Redundant random and fixed effects were removed from the initial model as described above. The final model included no random effects and the fixed factor participant group, revealing a significant main effect of participant group (*estimate* = −0.32, *SE* = 0.12, *z* = −2.73, *p* = 0.006), with significantly more N2 attachment choices for native compared to non-native speakers. The final model has a marginal R^2^_GLMM_ of 0.03, suggesting that only 3% of the variance in attachment preferences can be explained through the fixed effects in the model.

#### Response Times

**FIGURE 3:**
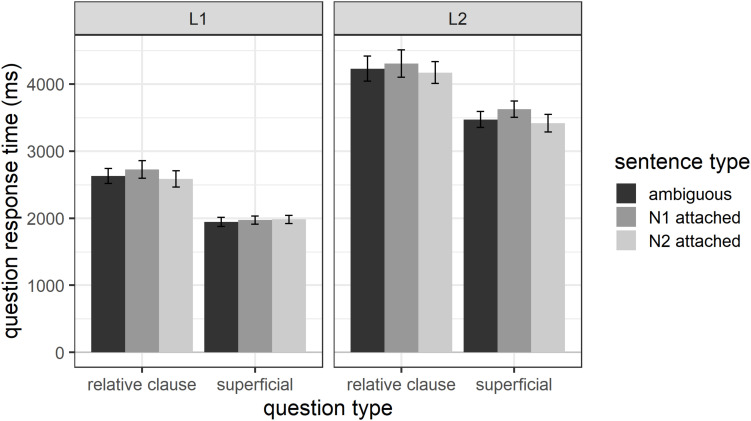
Question-response times as a function of question type, sentence type, and participant group.

Figure 3 shows the question-response times for both groups of participants and all sentence types and question types. In line with the response-accuracy data, the figure suggests that participants in the relative-clause group took longer to answer questions than participants in the superficial group. In addition, non-native speakers took numerically longer to respond to questions than native speakers. Question-response times were analyzed with linear mixed effect models. As before, the initial model included sentence type (N1 attached, N2 attached), question type (relative clause, superficial), participant group (L1, L2), and all interactions as fixed effects. The random effects structure included random intercepts for participant and item, and by-participant and by-item random slopes for the within-participant factor sentence type. Redundant random effects and fixed effects or interactions were removed from the initial model as described above.

The final model included random intercepts for participants and items and only the fixed factors question type and participant group. It showed a significant effect of question type (*estimate* = −355.29, *SE* = 94.71, *t* = −3.75, *p* < 0.001) with participants receiving superficial questions responding significantly faster than participants receiving relative-clause questions. In addition, there was a reliable effect of participant group (*estimate* = 780.28, *SE* = 94.70, *t* = −8.24, *p* < 0.001) with native speakers responding significantly faster than non-native speakers. The final model has a marginal R^2^_GLMM_ of 0.22 and a conditional R^2^_GLMM_ of 0.48, suggesting that 22% of the variance in question response times can be explained through the fixed effects in the model and 48% through the fixed and random effects.

### RC Attachment in Globally Ambiguous Sentences

The previous results for the native speakers receiving relative-clause questions are compatible with the idea that readers attach the relative clause during online processing. Reading times at the disambiguating region for the native speakers’ preferred N2-attached sentences are comparable to native speakers’ reading times for the globally ambiguous sentences. This suggests that native speakers could equally have made an attachment decision online when encountering an ambiguous reflexive pronoun, just as they did for the N2-attached sentences. However, the previous results leave us with the question of when the non-native speakers who received relative-clause questions attached the relative clause in globally ambiguous sentences. If the non-native speakers in this study attached the relative clause during online processing, i.e., at the ambiguous reflexive pronoun, then reading times should have been similar for globally ambiguous and disambiguated sentences for region 4, as the disambiguated sentences should reflect the time it takes to attach the relative clause. Instead, reading times were significantly faster for globally ambiguous compared to disambiguated sentences. This suggests that non-native speakers may have delayed their attachment decision until the comprehension question, and we should have seen longer question-response times for globally ambiguous compared to disambiguated sentences. However, there was no significant effect of sentence type for question-response times. So, when did non-native participants receiving relative-clause questions make attachment decisions for globally ambiguous sentences?

**FIGURE 4:**
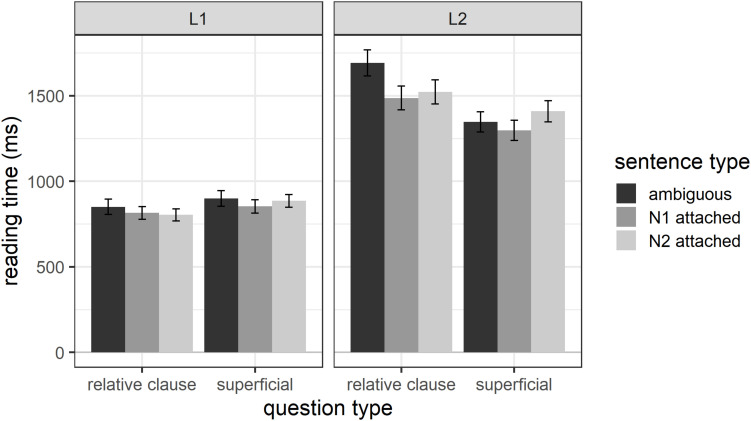
Reading times for region 7 as a function of question type, sentence type, and participant group.

In the following, we will explore the possibility that L2 participants may have attached the relative clause at the end of the sentence (when reading region 7), in anticipation of a relative-clause question. Outlier exclusion followed the same procedure as before and led to a total of 4.49% of data points being excluded. Overall, L1 participants had average reading times of 854 ms (*SD* = 504) and L2 participants of 1458 ms (*SD* = 850) for region 7. Figure 4 shows the region 7 reading times for each sentence type. For consistency with previous figures, Figure 4 shows reading times for region 7 for both L1 and L2 participants receiving relative-clause questions or superficial questions, but our analyses here focus solely on the L2 participants receiving relative clause questions. The figure shows that L2 participants receiving relative clause questions do in fact have numerically longer reading times for globally ambiguous compared to disambiguated sentences, a pattern we would expect if the non-native speakers attached the relative clause in anticipation of a question probing their attachment choice.

We thus conducted an analysis of reading times for Region 7 for the non-native speakers receiving relative-clause questions. The initial mixed effects model included sentence type (ambiguous, N1 attached, N2 attached), region length (number of characters), and their interaction as fixed effects. The random effects structure included random intercepts for participant and item, and by-participant and by-item random slopes for both fixed factors. The final model included random intercepts for participant and item and both fixed effects. The results showed a significant main effect of sentence type (*estimate* = −88.6, *SE* = 33.9, *t* = −2.62, *p* = 0.009). *Post hoc* tests using the emmeans() function showed that, as expected, sentence-final reading times were significantly longer for ambiguous sentences as compared to N1-attached (*estimate* = 215.2, *SE* = 83.0, *t* = 2.59, *p* = 0.027) and N2-attached sentences (*estimate* = 216.8, *SE* = 82.8, *t* = 2.62, *p* = 0.025). Reading times for N1- and N2-attached sentences did not differ significantly (*estimate* = 1.6, *SE* = 83.0, *t* = 0.02, *p* = 0.999). This suggests that non-native participants may have attached the relative clause in anticipation of a question probing their attachment choice.

There was also a significant main effect of region length (*estimate* = 298.8, *SE* = 47.1, *t* = 6.35, *p* < 0.001), again with longer regions taking longer to read. The final model has a marginal R^2^_GLMM_ of 0.10 and a conditional R^2^_GLMM_ of 0.36, suggesting that only 10% of the variance in reading times can be explained through the fixed effects in the model and 36% through the fixed and random effects.

## Discussion

### Task Effects

We found clear task effects in the current study. Specifically, our study replicates the main effect of question type found in Swets et al.’s (2008) study and thus extends this result to non-native speakers: L1 and L2 participants receiving superficial questions processed the disambiguating region reliably faster than participants receiving relative-clause questions. This result suggests that task demands modulate reading times for the reflexive pronoun in both native and non-native speakers, such that comprehension questions for target sentences that probe participants’ interpretation of the relative clause lead to slower processing of the reflexive pronoun than comprehension questions that can be answered without attaching the relative clause.

The main effect of question type is quite intriguing because participants receiving superficial questions received the same comprehension questions for filler sentences as participants receiving relative-clause questions. Thus, unlike Swets et al.’s (2008) study, the questions across groups only differed for target sentences. In light of this, it is notable that reading times for the disambiguating region differ substantially across question types. This suggests that the participants who took part in this study are quite sensitive to the task at hand. Even though filler questions were not superficial, participants were overall sensitive enough to the question manipulation to either expect or not expect questions probing relative-clause attachment.

While the above results are not necessarily incompatible with the shallow structure hypothesis (Clahsen and Felser, 2017) or Race (van Gompel et al., 2001), they are overall more easily integrated into a Good-Enough approach, which explicitly states that sentence processing is influenced by task demands. In particular, we do find shallow processing in both the native and non-native speakers in this study, but the shallow processing in both native and non-native speakers does not seem to result from an inability to process the sentences more deeply. If L2 participants were primarily guided by lexico-semantic and pragmatic information, i.e., if “the shallow processing route predominates” (Clahsen and Felser, 2006a, p. 117) in L2 participants, shallow parsing should prevail under both task conditions. Instead, similar to our results and Swets et al.’s (2008) results for native speakers, the shallow processing in non-native speakers seems to be strategic in that they engaged in shallow processing when deep processing was not required to accomplish the task.

Overall, the similarity of Swets et al.’s (2008) results and our results from native and non-native speakers in terms of task effects suggests that there is no *fundamental* difference in syntactic knowledge or sentence processing between native and non-native speakers. Thus, the shallow processing patterns we find here for non-native speakers are similar to those found for native speakers, and our results are most compatible with a Good-Enough approach to sentence processing.

As in Swets et al. (2008), participants in this study also took longer and were less accurate in responding to relative-clause questions compared to superficial questions. The lower accuracy rates for relative-clause questions may simply be due to these questions being harder than the superficial questions. The accuracy results from native speakers suggest that the relative-clause questions, with overall accuracy rates of about 76% in both Swets et al. and in the current study, are indeed more challenging than the superficial questions, which yielded accuracy rates of about 93% in Swets et al. and about 96% in the current study. The non-native speakers in our study were only slightly less accurate than the native speakers, with about 66% and 88% accuracy, respectively. In Good-Enough processing terms, these lower accuracy rates could be framed as the syntactic representations being less concrete and more prone to decay for non-natives (Ferreira, 2003; Lim and Christianson, 2013b).

Alternatively, the non-native speakers’ language background may explain their poorer performance for the relative-clause questions compared to the superficial questions. Chinese is a language that does not mark gender as a grammatical category. Chinese pronouns referring to male and female antecedents have been distinguished in writing since the early 20th century. However, they are not distinguished in speech. Pronouns referring to male and female antecedents are both pronounced as [ta] (Ettner, 2001). Dong et al. (2015) discussed the challenges for Chinese EFL learners in the production of gender agreement in English, noting that on average they produced a much higher error rate than Japanese and French EFL learners. Chen and Su (2011) found that Chinese speakers were less accurate at recalling the gender of a protagonist from a story they had listened to in their native language when compared with English speakers who were tested in a parallel experiment in English. Chinese word order may cause further difficulties for learners since the possessor is placed before the modifying NP in complex NPs involving a genitive construction (e.g., the actress PARTICLE-*de* sister, with *de* indicating the possessive). Thus, participants may be engaged in reordering the NP complex in the target items, possibly taxing the resources of less skilled readers. It is therefore possible that the lower accuracy rates for relative-clause questions reflect participants’ difficulties in matching the gender of the antecedent with the form of the reflexive pronoun. Feedback from participants during post-experiment interviews indicate that they did indeed struggle with correctly identifying potential antecedents for relative-clause disambiguation.

### Attachment Preferences

In line with many previous studies (e.g., Felser et al., 2003; Papadopoulou and Clahsen, 2003), the non-native speakers in our study did not show any clear attachment preferences. Such a result has often been interpreted as a failure to use syntactic parsing principles and taken as support of the shallow structure hypothesis (e.g., Dinçtopal-Deniz, 2010). Conversely, a propensity to attach a relative clause either high or low has served as counter evidence (e.g., Witzel et al., 2012). However, ours and Swets et al.’s (2008) results are internally consistent, such that differences in attachment preferences are reflected in differences in reading times both in the current study and in Swets et al. (2008). The question is therefore whether our non-native speakers’ lack of attachment preference in itself allows us to claim an inability to carry out syntactic operations.

In the case of this study, the non-native speakers’ native language background may provide an explanation for the lack of clear attachment preferences. An interesting feature of Chinese relative clauses is that they may be considered as a subset of noun modifying constructions, where no grammatical relationship exists between the head noun and the modifying clause (Chan et al., 2011). As such, their interpretations may depend on the semantics of the head noun and the hearer/reader’s world knowledge of the relationship between the head noun and the modifier. Chan et al. (2011) suggest that investigating Chinese relative clause processing may require an approach that emphasizes the role of semantics and pragmatics. This could imply that L1 Chinese speakers do not have any default preference for relative clause attachments, not because they are not able to employ syntactic parsing strategies, but because semantics and world knowledge may play a substantial role in their interpretation of relative clauses in the L1. Recent findings by Zhou et al. (2018) support this conclusion. Their study indicates that sentence plausibility rather than structure predicts reading times and accuracy when interpreting Chinese object and subject relative clauses.

Clahsen and Felser (2006b) also raised the possibility that non-native speakers may show no attachment preference because they take a complex noun phrase as a single entity, reflecting their thematic-based structuring strategy (see also Gilboy et al., 1995; Frazier and Clifton, 1997, for a similar proposal within the Construal framework). Under a Good-Enough approach, taking a complex noun phrase as a single entity does not reflect an inability to use syntactic parsing principles, but is in itself a syntactic processing strategy. When the complex noun phrase preceding the relative clause is taken as a single entity, the processor can store it as a whole and the heuristic parsing mechanism of the processing system provides a fast route to equilibrium. If the reflexive pronoun is compatible with only one of the nouns in the complex noun phrase, the processor needs to break up the merged entity in order to determine the antecedent, causing disequilibrium. In this case, the algorithmic processor takes over and resolves the disruption, which results in longer reading times. If the reflexive pronoun is compatible with both nouns of the complex noun phrase, equilibrium is never disrupted and the heuristic parsing mechanism can operate swiftly and with ease.

As mentioned above, the differences in reading time results for native compared to non-native participants are entirely compatible with differences in these groups’ attachment preferences and need not reflect qualitative differences in processing. While the native English speakers showed a low attachment preference and (numerically) faster reading times for low-attached sentences compared to high-attached sentences, non-native English speakers showed no attachment preference and comparable reading times for low- and high-attached sentences – with both patterns being internally consistent. Crucially, the non-native speakers processed the reflexive pronoun faster for globally ambiguous sentences compared to temporarily ambiguous sentences, suggesting that the non-native participants attached the relative clause online in the case of temporarily ambiguous sentences, whereas globally ambiguous sentences remained unattached. Thus, non-native speakers’ similar reading times for low- and high-attached sentences are both internally consistent and need not reflect an inability to engage in deep parsing, but instead may simply reflect that attaching relative clauses low vs. high takes equally long in the absence of any attachment preferences.

The reading time patterns that we found for globally ambiguous sentences across participant groups may also be related to attachment preferences. Specifically, L1 participants in Swets et al. (2008) and in our study processed the reflexive pronoun in the *preferred* low-attachment sentences as quickly as in the globally ambiguous sentences, reflecting faster processing when the preferred interpretation is possible compared to when it is not. In contrast, in the absence of clear attachment preferences, our non-native participants processed the reflexive pronoun in the globally ambiguous sentences faster than in both kinds of disambiguated sentences.

Our non-native results also extend earlier results from native speakers that ambiguous sentences can yield a processing advantage compared to disambiguated sentences (Traxler et al., 1998). In general, such findings support the Good-Enough approach and the Race model of sentence processing (e.g., van Gompel et al., 2001), but are incompatible with constraint-based models (e.g., MacDonald et al., 1994; Spivey-Knowlton and Sedivy, 1995), which predict longer reading times for globally ambiguous compared to disambiguated sentences. Race proposes that the parser is agnostic to whichever attachment option, and will adopt whichever “wins the race” to the end. Since both interpretations are compatible with globally ambiguous sentences, the adopted option will always be the final interpretation since it does not conflict with the reflexive pronoun. In contrast, only one option is allowed in temporarily ambiguous sentences; if the wrong noun phrase is adopted early in the parse, it will have to be abandoned later on, incurring greater processing costs. In contrast, Good-Enough processing assumes that relative clauses may remain unattached, leading to their faster reading times for globally ambiguous sentences relative to disambiguated sentences. Thus, both Good-enough processing and Race are compatible with faster reading times for globally ambiguous sentences compared to disambiguated ones.

Similar to Swets et al. (2008), we also found evidence for delayed attachment in relative clause processing. This supports a Good-Enough rather than a race-based approach to sentence processing since only Good-Enough processing allows for delayed attachment of relative clauses. Specifically, Swets et al. (2008) found longer response times for relative-clause questions following globally ambiguous sentences compared to temporarily ambiguous sentences, and suggested that these longer question-response times for globally ambiguous sentences reflect participants attaching the relative clause not online, but when forced to do so by a relative-clause comprehension question. In contrast, we find evidence for a similar process not in our L1, but in our L2 participants, who delayed their attachment decision for globally ambiguous sentences until the end of the sentence, in anticipation of a relative-clause comprehension question. Unlike participants in Swets et al.’s study, the native participants in the current study seemed to attach the relative clause online, even for globally ambiguous sentences.

The non-native participants in the current study may have delayed their attachment decision for globally ambiguous sentences until the end of the sentence to prolong a state of equilibrium, possibly because they do not have a preferred attachment option. Similarly, L1 participants receiving relative-clause questions in our study may not have delayed their attachment decision because they do have a preferred attachment option. Attaching the relative clause may cause less disruption of the equilibrium when the attachment decision is easy, as in the case of having a preferred attachment choice.

Alternatively, the above-mentioned difficulties that native Chinese speakers have in identifying antecedents may be the reason why non-native participants, but not native participants, in the current study initially left globally ambiguous sentences unattached and delayed their attachment decision. Despite these difficulties, however, non-native participants did attempt to identify potential antecedents online when needed, as reflected in the longer reading times for the reflexive pronoun in the disambiguated compared to globally ambiguous conditions.

Finally, we suggest that the results from the current study point to quantitative rather than qualitative differences in L1 and L2 sentence processing. In particular, there are quantitative differences between the native and non-native participants in terms of overall reading times, response accuracy and response times, with non-native participants showing significantly slower reading times and response times as well as lower response accuracy across the board. Thus, non-native participants take longer to read the sentences and are less accurate in responding to comprehension questions, but their overall reading time and response *patterns* are internally consistent and suggest no qualitative differences from native speakers.

## Conclusion

This study explored the Good-Enough approach in native and non-native sentence processing by investigating whether native English speakers and native Chinese L2 learners of English adjusted their parsing strategies to meet the demands of the task at hand. Despite an absence of clear attachment preferences, the non-native speakers in this study processed globally and temporarily ambiguous sentences similarly to native speakers and showed clear effects of both sentence type and task demands on sentence processing. Overall, the results support the Good-Enough approach to sentence processing and suggest no *fundamental* difference between native and non-native sentence processing. Thus, we propose that rather than assume a different treatment of shallowness in native and non-native processing, the Good-Enough account of sentence processing and its task-based approach to shallowness can be extended to non-native processing.

## Data Availability Statement

The datasets presented in this study can be found in online repositories. The names of the repository/repositories and accession number(s) can be found below: The data and scripts are available at the Open Science Framework at https://osf.io/4szpx/.

## Ethics Statement

The studies involving human participants were reviewed and approved by the Ethics committee of the College of Arts and Humanities (now the College of Arts, Humanities and Business) at Bangor University (Approval Nos. LX-1423 and LX-1589). The patients/participants provided their written informed consent to participate in this study.

## Author Contributions

The L2 data for the study come from MT’s Master’s Thesis, supervised by AF. MT designed the experiment and ran the L2 participants and wrote the introduction. AF provided feedback on the design and ran the L1 participants. Both authors did the data analysis and co-wrote the other parts of the manuscript.

## Conflict of Interest

The authors declare that the research was conducted in the absence of any commercial or financial relationships that could be construed as a potential conflict of interest.
